# The efficacy and safety of cupping therapy for treating of intractable peripheral facial paralysis: A protocol for systematic review and meta-analysis

**DOI:** 10.1097/MD.0000000000025388

**Published:** 2021-04-23

**Authors:** Zhiwen Cao, Lin Jiao, Hongyu Wang, Jun Li, Genping Zhong, Daocheng Zhu, Wei Xu, MengKe Jin

**Affiliations:** a Jiangxi University of Traditional Chinese Medicine; b The Affiliated Hospital of Jiangxi University of Traditional Chinese Medicine, Nanchang, China.

**Keywords:** cupping, intractable facial paralysis, meta-analysis, systematic review

## Abstract

**Background:**

Peripheral facial paralysis (PFP) is a common clinical neurological disease and the incidence of intractable peripheral facial paralysis is on the rise. Symptoms include crooked mouth and eyes, tearing and shallow nasolabial folds. The disease seriously affects the physical and mental health of patients. At present, a large number of clinical studies have shown that cupping is effective in treating intractable peripheral facial paralysis (IPFP). Therefore, the purpose of this review is to evaluate the effectiveness and safety of cupping in the treatment of refractory peripheral facial paralysis.

**Methods:**

We will conduct a comprehensive and systematic search of relevant documents in the following databases: Medline, PubMed, the Cochrane Central Register of Controlled Trials (CENTRAL), Embase, Chinese Biomedical Literatures Database, China National Knowledge Infrastructure (CNKI), Wang Fang Database, Chinese Scientific Journal Database from inception to February 2021 without any language restriction. The 2 reviewers will be independently completed select research, extract data, evaluate research quality and use the Cochrane risk of bias tool to assess methodological quality. Using revman5.4 software for statistical analysis. The degree of heterogeneity will be Determined through heterogeneity test, to definite whether to adopt a random effects model or a fixed-effects model.

**Results:**

The protocol for the meta-analysis will systematically evaluate the efficacy and safety of cupping therapy for intractable peripheral facial paralysis patients.

**Conclusion:**

This study will explore whether or not cupping therapy can be used as one of the non-drug therapies to prevent or treat intractable peripheral facial paralysis

## 1 Introduction

Peripheral facial paralysis (PFP) refers to a type of disease that causes of partial or complete loss of facial nerve function due to damage to lower motor neurons (LMN) of the 7th cranial nerve.^[1]^ The disease usually starts acutely, which manifested as unilateral muscle palsy, crooked mouth angle to the healthy side, obliteration of the naso-labial fold, shallowing or disappearing off the frontal lines on the affected side, incomplete eyelid closure, tearing, inability to raise the eyebrows and wrinkle the forehead, poor whistling and pain behind the ear, some patients may have weakened taste and hyperacusis.^[2]^ In clinic, intractable facial paralysis is considered to be a facial paralysis that has a course of >2 months, but still has facial sequelae symptoms.^[3]^

Bell palsy, named as idiopathic facial paralysis, is the most common one in clinic, and it does not have identifiable causes.^[4]^ Although many cases are idiopathic, others are associated with identifiable causes, such as otitis media, local trauma to the, sarcoidosis, postsurgical complications, facial nerve, neoplasms, or reactivated varicella zoster virus infection of the geniculate ganglion (Ramsay Hunt syndrome).^[4]^ According to a report, 37 people per 100,000 people suffer from PFP every year.^[5]^ American scholars Morales and Donnan found that more than 60,000 sicks are infected with Bell palsy every year.^[6]^ In addition, the incidence varies among age groups, the annual incidence of adult peripheral facial paralysis attractions is about 17∼35 per 100,000,^[7]^ whereas the incidence is higher in patients over the age of 70.^[8]^ A research found that one in 60 people with the disease is at risk of death,^[9]^ and two-thirds of patients suffer from abnormal regeneration of the facial nerve, with chronic sequelae.^[10,11]^ Facial expression plays a significantly important role in interpersonal communications, and facial paralysis severely hinders this function.^[12]^ Studies have shown that up to 30% of patients with PFP have poor recovery of facial muscle control, facial deformities, facial pain, and thus suffer great psychological trauma.^[13,14]^ Furthermore, PFP also can increase the risk of cardiovascular disease, depression, anxiety disorders, and so on.^[15–17]^ Therefore, there is an urgent need for an effective therapy to help patients relieve pain and medical stress.

At present, the treatment of peripheral facial paralysis mainly includes drugs (corticosteroids, antiviral drugs) and surgery.^[18–22]^ However, taking medicines will bring some side effects. Adverse events of corticosteroids include osteoporosis, cardiovascular disease, impaired immune response and wound healing, changes in glucose and lipid metabolism, and mental disorders.^[23]^ Besides, some adverse effects can be witnessed in patients taking antiviral drugs, which include digestive symptoms, such as nausea, vomiting, and diarrhea, and neurological symptoms, such as dizziness and convulsions (more common at high doses).^[4]^ What is more, the high cost of surgery discourages some families. Therefore, facial paralysis patients urgently need a safe and effective alternative therapy to relieve the pain caused by the disease.

PFP belongs to “skewed mouth and eyes,” “hanging line wind,” and “skewed mouth wind” in Traditional Chinese Medicine (TCM).^[24]^ Based on the TCM, The pathogenesis is mostly caused by Pathogenic wind-cold or wind-heat, which taking advantage of the deficiency to invade Yangming and Shaoyang meridian. The emptiness of meridian causes the movement of the qi obstructed, the circulation of qi and blood runs out of balance and the muscles are abnormality.^[25]^ In fact, the veins’ emptiness is the internal cause, and the and the invasion of evils is the external cause, the internal and external factors interact with each other to cause the disease.

Cupping therapy is considered as nonmedicinal external treatment of TCM, which has a history of >2000 years,^[26]^ and the earliest ancient records about cupping therapy can be dated back to Prescriptions of *Fifty-two Diseases* from the period of the Tang Dynasty.^[27]^ In the past, cupping was also called “cupping gas” or “horn cups” (made of ox horn) in ancient times.^[28]^ And the principle is to use cups as the treatment medium, and use methods such as burning oxygen or sucking air to make the cups present a negative pressure state. Using this physical principle, it is adsorbed to the corresponding meridian points of the human body or a certain part, Cause local blood stasis.^[29]^ As a green and convenient medical tool, cupping has been widely used all over the world.^[30]^ At present, cupping therapy has been widely used in the treatment of IPFP in my country, based on literature studies, it has been found that cupping therapy has a significant effect on facial nerve palsy.^[31–33]^ Ting Li, Laotian Li use CW-NIRS to evaluate blood oxygen changes during cupping therapy treatment, study has found that cupping can reduce the patient's deoxygenated hemoglobin, obtain more oxygenated hemoglobin, enhance local oxygen injection, promote blood microcirculation and hemodynamic activity, accelerate the possible repair or function of local tissues, and produce positive therapeutic effects.^[34]^

Although the effects of cupping have been widely confirmed, the effectiveness and safety of cupping in the treatment of intractable peripheral facial paralysis are still controversial. Systemic review or meta-analysis has been considered as the basis for evaluating clinical efficacy and formulating clinical guidelines.^[35,36]^ Therefore, this study adopts evidence-based medicine to systematically analyze and evaluate randomized controlled trials (RCTs) in the treatment of intractable peripheral facial paralysis patients with cupping to provide a basis for verifying the efficacy.

## 2 Methods and analysis

### 2.1 Study registration

This protocol was registered on the International Platform of Registered Systematic Review and Meta-Analysis Protocols (INPLASY) on February 19, 2021 (registration number INPLASY202120062). You can check its authenticity on this website (https://inplasy.com/inplasy-2021-2-0062/). We will strictly perform this protocol by following the Preferred Reporting Items for Systematic Reviews and Meta-Analyses Protocol (PRISMA-P) statement guidelines.^[37]^

### 2.2 Eligibility criteria

#### 2.2.1 Type of studies

The RCT on cupping treatment of patients with refractory peripheral facial paralysis will be fully searched in the Chinese and English databases. There are no restrictions on language and publication status. In addition, non-RCTs must be excluded.

#### 2.2.2 Types of participants

The inclusion of this literature must be RCT. The inclusion cases must meet the diagnostic criteria of peripheral facial paralysis, and the course of disease are >2 months. This article does not limit the age, sex, and source of the patient. Patients with other diseases in patients with chronic cholecystitis will be excluded.

#### 2.2.3 Types of interventions

Patients with intractable facial paralysis in the test group must be treated with cupping therapy as the main regimen (either in combination with other treatments or alone) and the control group must be treated with non-cupping therapy.

#### 2.2.4 Type of comparators

The control group can include blank control, medicine (traditional Chinese medicine, western medicine) treatment, conventional symptomatic treatment, among others.

1. cupping therapy versus no treatment;
2. cupping therapy versus placebo;
3. cupping therapy versus symptomatic or active treatment;

#### 2.2.5 Types of outcome measures

##### 2.2.5.1 Primary outcomes

The total effective rate

##### 2.2.5.2 Secondary outcomes.

1. House-Brickman facial nerve function classification
2. Sunnybrook Facial Nerve Rating System Scale
3. The Facial Disability Index (FDI) scale
4. Recurrence rate
5. Adverse events

### 2.3 Exclusion criteria

No matter before or after the research, cupping is the only control group, excluding repetitive research, animal experiments such as mice or rabbits, literature review, theoretical discussion, nursing experiment, and so on.

### 2.4 Search strategy

Eight electronic databases including PubMed, Web of Science, Cochrane Database of Systematic Reviews, EMBASE, Chinese Biomedical Literatures Database, China National Knowledge Infrastructure, Wang Fang Database, Chinese Scientific Journal Database will be searched without any language restriction from their inception to February 2021. The search uses the keyword search. The main subject terms searched: “cupping,” “facial paralysis.” The retrieval strategy of PubMed database is shown in Table 1. The search strategies of major databases will be adjusted according to the different databases.

**Table 1:** The search strategy for Pubmed.

Search Strategy (PubMed)	
Order	strategy
#1	Search “Facial Paralysis”[Mesh]
#2	Search “Paralyses, Facial” [Title/Abstract] or “Paralysis, Facial” [Title/Abstract] or “Facial Palsy”[Title/Abstract] or “Facial Palsies” [Title/Abstract] or “bell pasly” [Title/Abstract] or “facial nerve paralysis” [Title/Abstract] or “Peripheral facial paralysis ” [Title/Abstract] or “facial nerve paralysis” [Title/Abstract] “Lower Motor Neuron Facial Palsy”
#3	#1 OR #2
#4	Search “Cupping”[Mesh] OR “Cupping Therapy”[Mesh]
#5	Search “Cupping Therapies^∗^” [Title/Abstract] or “Therapy, Cupping^∗^” [Title/Abstract] or “Cupping Treatment”[Title/Abstract] or “Cupping Treatments ” [Title/Abstract] or “Treatment, Cupping” [Title/Abstract]
#6	#4 AND #5
#7	Search “intractable” [Title/Abstract]or “refractory”
#8	Search “Randomized controlled trial” [MeSH] or “controlled clinical trial” [MeSH]
#9	Search “Randomized controlled trial” [Title/Abstract] or “clinical trial” [Title/Abstract] or “randomized” [Title/Abstract]
#10	#8 OR #9
#11	#3AND#6AND#7AND#10

### 2.5 Process of selection

#### 2.5.1 Screening steps

**Figure 1:**
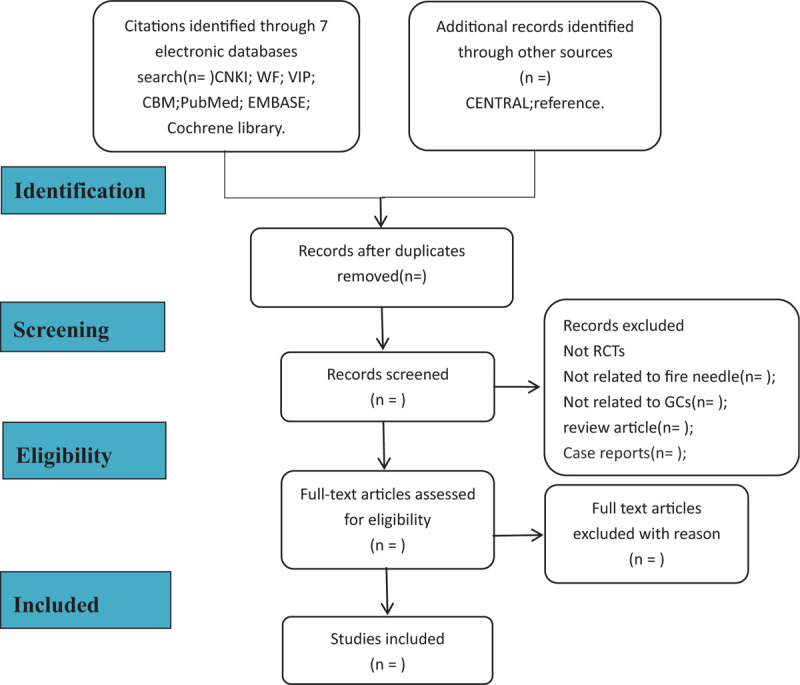
Flowchart of literature selection.

We will process the files that can finally be used according to certain operating procedures; first, we retrieve all the documents needed from the database according to the correct subject terms and import them to the document manager using the correct import method in NoteExpress 3.0. In the document manager, delete duplicate documents; then, by reading the title and abstract, delete documents that are not related to this system review; download the remaining articles one by one and read the full text; finally, according to the various standards discussed above Determine the final document. For this operation, 2 researchers (Hongyu, Wang, Wei, Xu) strictly follow this procedure. If they disagree, consult the third evaluator (ZC) for negotiation. The included article process is shown in Figure 1.

#### 2.5.2 Information extraction

During the article data collection process, 2 researchers (ZC, HW) will independently extract the required information and input it into Word2010. When the information is about to be entered, cross-check the information extracted by the 2 researchers. If you have any questions about the included literature, you can contact the third reviewer (CZ) for consultation to ensure the accuracy of the article. The extracted information includes: title, author, publication year, sample size, intervention measures, and outcome index, and so on. If important information is missing from the information entered in the article, please contact the author of the article by phone or email.

#### 2.5.3 Methodological quality evaluation

The article quality evaluation and bias risk assessment methods were carried out using Cochrane Reviewer's Handbook 5.0. The content includes: random method; allocation hiding; realization of blind method; completeness of the result data; whether the scorer is blinded or not; selective reporting of results; other errors. The above 7 items all include “yes,” “no,” and “no.” “Clear” 3 options, 2 evaluators need to evaluate the options that meet the conditions. If there is a dispute between the 2 parties during the selection process, they can consult a third party (ZC) for handling.

### 2.6 Data synthesis

#### 2.6.1 Quantitative data analysis

Meta-analysis was performed using RevMan5.4 provided by the Cochrane collaboration network. Odds ratio (OR) and 95% confidence interval (CI) will be used in enumeration data; measurement data are represented by weighted mean difference (WMD) and 95% CI, or standardized mean difference (SMD) should be used when the units were not unified

#### 2.6.2 Heterogeneity analysis

The heterogeneity test between the results of the included studies was performed using the *I*^2^ test. The *I*^2^ value reflects the proportion of the total variation in the effect size due to the existence of heterogeneity. (*I*^2^ > 50%), indicating that heterogeneity is more obvious. If there is no obvious heterogeneity between the research results (*I*^2^ < 50%), the fixed-effect model is used to merge them; if there is significant heterogeneity (*I*^2^ > 50%), the source of the heterogeneity is analyzed first, which may lead to heterogeneity Factors for subgroup analysis.

#### 2.6.3 The publication bias

Since there is a large amount of literature on cupping treatment of refractory facial paralysis, Review Manager 5.4 inverted funnel chart is used for bias analysis to find out the source of heterogeneity.

#### 2.6.4 Subgroup analyses

Subgroup analysis will be handled according to the differences in cupping methods, patient conditions, and control.

#### 2.6.5 Sensitivity analysis

Sensitivity analyses will be performed to verify the robustness of the review conclusions. Analysis software uses STATA 14.0 software for sensitivity analysis.

## 3 Discussion

Based on the current modern clinical curative effect research, this article reviews the safety and effectiveness of acupuncture and moxibustion in the treatment of refractory facial paralysis for the first time. The purpose is to find favorable evidence-based evidence for the application of cupping therapy in refractory facial paralysis. According to the discussion part of this article, the following aspects will be elaborated:

1. TCM pathogenesis of intractable peripheral facial paralysis.
2. The advantages and disadvantages of cupping therapy in the treatment of intractable peripheral facial paralysis and the mechanism of acupuncture and moxibustion.
3. Horizontal comparison with other treatment methods (Chinese and Western medicine, surgery, among others).
4. Explain the results of this evaluation.
5. Conclusion.

Intractable peripheral facial paralysis occurs at any age, and the disease brings both physical and psychological damage to patients. Cupping is an effective nonmedicinal treatment of traditional Chinese medicine and has been widely used by doctors at home and abroad. In China, cupping therapy has been widely used in the treatment of intractable peripheral facial paralysis. However, the current cupping therapy has no systematic scientific evaluation on intractable peripheral facial paralysis, so the purpose of this article is to testify cupping. Therefore, this overview aims to provide real and reliable research evidence for the treatment of intractable peripheral facial paralysis With cupping. Of course, this article still has many shortcomings. First, the quality of the included literature is not high, which has an adverse effect on the article. Second, differences in Chinese and English languages, and differences in the course of the included cases affect the credibility of the article. Third is the failure to contact the author of the article, resulting in incomplete article data. Therefore, for more high-quality RCTs and research mechanisms are needed to confirm its effectiveness, so as to more objectively evaluate the safety and effectiveness of cupping in the treatment of intractable peripheral facial paralysis.

## Author contributions

**Conceptualization:** Zhiwen Cao.

**Data curation:** Zhiwen Cao, Hongyu Wang

**Formal analysis:** Zhiwen Cao, Jun, Li

**Funding acquisition:** Lin Jiao.

**Methodology:** Zhiwen Cao, Mengke Jin

**Project administration:** Lin Jiao

**Software:** Zhiwen Cao, Wei Xu.

**Validation:** Lin Jiao

**Writing – original draft:** Zhiwen Cao, Hongyu Wang, Daocheng, Zhu

**Writing – review & editing:** Mengke Jin, Genping Zhong, Daocheng, Zhu
